# Evaluation of perioperative pachymetry of thin corneas during corneal crosslinking using hypo-osmolar riboflavin and hydroxypropyl methylcellulose

**DOI:** 10.5935/0004-2749.2023-0056

**Published:** 2024-07-09

**Authors:** Fábio Kenji Matsumoto, Marcelo Tojar, Luciene Barbosa de Souza

**Affiliations:** 1 Department of Ophthalmology, Universidade Federal de São Paulo, São Paulo, SP, Brazil

**Keywords:** Corneal pachymetry, corneal topography, cross-linking reagents/therapeutic use, hypromellose derivatives, keratoconus/surgery, riboflavin/therapeutic use

## Abstract

**Purpose:**

This study aimed to analyze variations in intraoperative corneal thickness during corneal cross-linking in patients with keratoconus and to investigate its possible correlation with presurgical maximal keratometry (Kmax) and pachymetry.

**Methods:**

This was a prospective case series. We used a method similar to the Dresden protocol, with the application of hydroxypropyl methylcellulose 0.1% hypo-osmolar riboflavin in corneas between 330 and 400 µm after epithelium removal. Corneal thickness was measured using portable calipers before and immediately after epithelium removal, and 30 and 60 min after the procedure.

**Results:**

The 30 patients in this study were followed up for one year. A statistically significant difference was observed in pachymetry values during the intraoperative period (p<0.0001) and an increase of 3.05 µm (95%C1: 0.56-5.54) for each diopter was seen after epithelium removal (p0.019). We found an average Kmax difference of —2.12 D between men and women (p0.013). One year after treatment, there was a statistically significant reduction in pachymetry (p<0.0001) and Kmax (p0.0170) values.

**Conclusions:**

A significant increase in pachymetry measurements was seen during the procedure, and most patients showed a regression in Kmax and pachymetry values one year after surgery.

## INTRODUCTION

Keratoconus is among the more common corneal ectasia diseases. Risk factors include a hereditary predisposition, chronic scratching, inflammation, atopy, poorly adapted contact lenses, and oxidative stress. Patients with a family history of keratoconus have a 15-67 times greater risk than those without^(1)^. However, keratoconus is a multifactorial disease and other factors are necessary for its development^(2)^.

In patients with keratoconus, the collagen fibers of the corneal stroma are elongated due to the stress caused by the protrusion of the cone^(3)^. 1n addition, the diameter of these fibers in the anterior, middle, and posterior stroma is smaller than those of individuals without keratoconus^(4)^. A decrease is also seen in the interfibrillar spacing between collagen fibers and proteoglycans^(5)^.

Studies of with keratoconus cases with corneal thicknesses of less than 400 µm have found an increased risk of endothelial damage by ultraviolet light type A (UVA) light. In such cases, hypo-osmolar riboflavin is used to induce swelling of the corneal stroma after its instillation^(6,7,8)^.

There has been considerable research into variations found in corneal cross-linking (CXL) patients. In this study, we analyze variations in the perioperative corneal thickness of thin corneas using only hypo-osmolar riboflavin with hydroxypropyl methylcellulose (HPMC). The patients were followed up for one year to observe the changes in maximal keratometry (Kmax) and pachymetry measurements, and to determine any correlations between these parameters and age, sex, or presurgical values.

## METHODS

This prospective case series was conducted at the External Eye Disease and Cornea Service in the Department of Ophthalmology of the Federal University of Sao Paulo (UN1FESP). All patients underwent a complete eye examination, specular microscopy (Nidek CEM 530 specular microscope®), and corneal tomography (Oculus Pentacam® HR). Hypo-osmolar riboflavin with HPMC 0.1% was used (200 mOsm). Pachymetry was performed using a portable Tomey SP-100® and the source of UVA was an Opto CXL laser® (370 nm; 3 mW/cm^2^; b5.4 J/cm^2^; 5 cm distance). All patients were followed up postoperatively for one year.

The procedure consisted of 8 mm of central epithelium removal. We instilled one drop of 0.1% riboflavin with 1% HPMC diluent without blepharostat every 5 min for 30 min during the corneal soaking phase. The solution was administered with blepharostat during the UVA irradiation phase at the same rate of one drop every 5 min for 30 min. Corneal thicknesses were measured using portable calipers (Tomey SP-100®) before and immediately after epithelium removal, and then 30 min and 60 min after the procedure.

The inclusion criteria for this study were age ≥14 years, a 0.75 D Kmax increase over 6 months or less or of 1.00 D in one year or less (maximum value of 70 D), an epithelium-free pachymetry measurement between 330 and 400 µm (epi-off), and no contact lens use for at least 14 days before the measurements and procedure. We excluded cases in which we expected visual improvement and those with acute hydrops keratoconus, significant corneal opacity, a corneal endothelial count <1000 cells/mm^2^, collagen, autoimmune or other systemic diseases, or pregnancy.

Data were analyzed using Stata v. 14.0 (StataCorp LP, College Station, TX, USA) software. The relationships between pre and post-treatment and covariate differences were investigated using multiple linear regression. 1ntraoperative variations in pachymetry values were evaluated using the Friedman test, followed by post-hoc analysis with the Dunn test. Results were considered significant when p-values were <0.05.

## RESULTS

This study included 30 patients (30 eyes), all of whom were followed up for one year. There were no complications in any of the patients at any point during the study. Most of the patients undergoing the procedure were male (14 eyes), aged 15-44 years (mean, 22.50 [±6.80]), and did not use contact lenses. The majority of the included eyes were the left eye (16 eyes).

The average thickness gain between the beginning and end of surgery was 164.57 (±40.72) µm, with a range of 98-261 µm (median, 160.50). This was a statistically significant difference (p<0.0001) (Table 1).

**Table 1:** Perioperative corneal thickness variation during corneal cross-linking

Pachymetry	Preoperative	Epithelium removal	30 min postoperative	60 min postoperative	Friedman Test
Mean (±SD) (median) µm	417.07 (±16.24) (420.50)	370.30 (±19.01) (373.50)	497.27 (±30.29) (499.50)	581.63 (±37.06) (578.00)	**p<0.001**

SD= standard deviation.

Regression analysis showed gender, age, and preoperative pachymetry values to be unrelated to the amount of intraoperative thickness gain (p>0.05) (Table 2). Preoperative Kmax values were significantly associated with intraoperative thickness gains, which increased by 3.05 µm (95%C1: 0.56 to -5.54) per diopter after epithelium removal in each respective patient (p = 0.019).

**Table 2:** Multiple linear regression for corneal thickness gains from corneal cross-linking adjusted for gender, age, and preoperative Kmax and pachymetry values

Pachymetry		
Coefficient (95%CI)		p-value
Sex		
Female	Reference	------
Male	−9.96 (−41.98-22.06)	0.529
Age	−0.12 (−2.21-1.97)	0.906
Preoperative Kmax	3.05 (0.56-5.54)	**0.019**
Preoperative pachymetry	−0.66 (−1.65-0.33)	0.18

Cl= confidence interval.

One year after treatment, there was a statistically significant reduction in the average pachymetry (p<0.0001) and Kmax (p=0.0170) values. There was no significant difference between pre and post-treatment specular microscopy values (p>0.05) (Table 3).

**Table 3:** Corneal values preoperatively and at one year follow-up of patients with thin corneas treated with corneal cross-linking

	Preoperative	One year follow-up	Difference	p-value
	Mean (±SD) (median)	Mean (±SD) (median)		
Pachymetry (µm)	414.83 (±17.66) (418.50)	390.37 (±20.03) (384.00)	−24.47 (±15.08) (−27.00)	**<0.0001**
Kmax (D)	61.12 (±5.61) (61.70)	60.45 (±5.56) (60.05)	−0.67 (±2.31) (−0.45)	**0.0170**
Specular microscopy (cells/mm^2^)	2698.93 (±279.90) (2681.50)	2643.70 (±280.35) (2612.00)	−55.23 (±155.09) (−6.50)	0.2134

SD= standard deviation.

The results of the regression analyses shown in table 4 indicated that gender, age, and pretreatment measurements were unrelated to the differences in pachymetry and specular microscopy (p>0.05) findings at one year follow-up. A difference of −2.12 D was found in the average Kmax values of men and women (p=0.013).

**Table 4:** Multiple linear regressions for the pre to postoperative differences in corneal values in corneal cross-linking patients adjusted for gender, age, and preoperative values

	Pachymetry (µm)	Pachymetry (×m)	Kmax (D)	Kmax (D)	Specular Microscopy (cells/mm^2^)	Specular Microscopy (cells/mm^2^)
	Coefficient (95%)	p-value	Coefficient (95%)	p-value	Coefficient (95%)	p-value
Sex						
Female	Reference	------	Reference	------	Reference	------
Male	−4.50 (−16.52-0.53)	0.449	−2.12 (−3.77-0.48)	**0.013**	66.74 (−62.60-96.07)	0.299
Age	−0.45 (−0.31-0.41)	0.295	−0.05 (−0.17-0.07)	0.362	−3.13 (−11.92-5.65)	0.47
Preoperative measurements	−0.22 (−0.56-0.11)	0.183	−0.10 (−0.24-0.04)	0.162	−0.11 (−0.34-0.11)	0.318

## DISCUSSION

There have been few studies in the scientific literature on this topic. Hafezi et al. conducted a similar study using isosmolar riboflavin first and then hypo-osmolar riboflavin to increase corneal thickness. As their procedure differed from ours, their results did also^(8)^.

We found the pachymetry value gains to vary quite widely between patients, and this was unrelated to age, preoperative pachymetry values, and sex. Multiple linear regression showed an increase in Kmax of 3.05 µm every 01 D from 51.5 D. Therefore, corneas with greater curvature gained more corneal thickness during the procedure.

This initial curving can be explained by the greater fragility of corneal biomechanics induced in the corneal stroma by the CXL procedure. This includes a 5% increase in collagen fiber diameters, fewer lamellae, and more degenerated fibrils.

There was an average decrease in pachymetry measurements at the one year follow-up, possibly due to the considerable reduction in the number of fibrils (8%-12%), even with the increased collagen fiber diameters (5%) and the absence of interfibrillar spacing^(8)^.

This study had some limitations, including a small sample size and significant variability in intraoperative pachymetry. Future studies should measure the corneal thickness in each case, especially those with smaller pachymetry values, to avoid possible endothelial injuries.

The results of this study indicate that CXL using hypo-osmolar riboflavin and HPMC is safe and effective in patients with thin corneas, with significant intraoperative increases in pachymetry values. There have been no complications in the patients to date. There was also a reduction in the Kmax and pachymetry measurements of most patients at their one year follow-up.
